# On the need for a new generation of coastal change models for the 21^st^ century

**DOI:** 10.1038/s41598-020-58376-x

**Published:** 2020-02-06

**Authors:** Roshanka Ranasinghe

**Affiliations:** 1 Department of Water Science and Engineering, IHE-Delft P.O. Box 3015, 2610 DA Delft, The Netherlands; 2 Harbour, Coastal and Offshore Engineering, Deltares, PO Box 177, 2600 MH Delft, The Netherlands; 3 Water Engineering and Management, Faculty of Engineering Technology, University of Twente, PO Box 217, 7500 AE Enschede, The Netherlands

**Keywords:** Natural hazards, Engineering

## Abstract

The combination of climate change impacts, declining fluvial sediment supply, and heavy human utilization of the coastal zone, arguably the most populated and developed land zone in the world, will very likely lead to massive socio-economic and environmental losses in the coming decades. Effective coastal planning/management strategies that can help circumvent such losses require reliable local scale (<~10 km) projections of coastal change resulting from the integrated effect of climate change driven variations in mean sea level, storm surge, waves, and riverflows. Presently available numerical models are unable to adequately fulfill this need. A new generation of multi-scale, probabilistic coastal change models is urgently needed to comprehensively assess and optimise coastal risk at local scale, enabling risk informed, climate proof adaptation measures that strike a good balance between risk and reward.

## Introduction

With approximately 10% of the global population living in the coastal zone^1^, the potentially massive impact of climate change on the world’s coastal zones is now well recognized^2–6^. Moreover, continued human attraction to the coast has resulted in rapid expansions in settlements, urbanization, infrastructure, economic activities and tourism, as exemplified by 15 of the world’s 20 megacities being located in the coastal zone^7^. The combination of climate change impacts, declining fluvial sediment supply^8,9^, and the ever increasing human utilization of the coastal zone is very likely to result in unprecedented socio-economic and environmental losses in the coming decades^10–15^. For example, the economic losses due to flooding alone in coastal cities is expected to be around US $ 1 Trillion by 2050^16^. Similarly, the cost of forced migration due to just sea level rise (SLR) driven coastal erosion over the 21^st^ century is also expected to be around US $ 1 Trillion^2^. Effective coastal planning/management strategies that can help circumvent such losses through adaptation require reliable projections of coastal change. This perspective addresses the question of how we may obtain such projections using numerical modelling techniques.

## Climate Change Driven Coastal Change

IPCC AR5 projections of globally averaged sea level rise (SLR) range from 0.26 m to 0.98 m (by 2081–2100 relative to 1986–2005)^17^. Future storms are expected to become more intense while average (or mean) wave conditions are also expected to be modified by climate change^17–19^. Storm surge intensity and frequency are both expected to change in future^6,17,20^, while riverflows are expected to change by up to 40% in some regions^21^. These climate change driven variations in natural forcing are likely to result in significant morphological impacts along, especially, the sandy coastlines of the world^3,5,22,23^, which constitute 31% of the global coastline^24^ and are subject to a very high level of human utilisation^2,25^. The potential first order climate change driven morphological impacts on sandy coasts, are summarized in Table 1, together with their main drivers and manifestation time scales.

**Table 1:** Potential first order climate change driven morphological impacts on sandy coasts (modified from^5^).

Potential impact	Process time scale*	Main drivers
Increased/decreased episodic storm erosion of beaches and dunes	Episodic	Changes in intensity and/or frequency of storms, changes in storm surge, changes in storm wave characteristics
More/less frequent (or previously unexperienced) episodic formation and closure of small tidal inlets	Episodic	Changes in storm surge, changes in intensity/frequency of extreme riverflow events, changes in storm wave characteristics
More/less breaching of Barrier islands	Episodic	Sea level rise, changes in intensity and/or frequency of storms, changes in storm surge
Sustained erosion/accretion due to permanent re-alignment of embayed beaches	Medium-term	Changes in mean offshore wave direction, changes in storm wave characteristics
More/less elongation of (updrift) barrier islands and subsequent changes in barrier inlet geometry	Medium-term	Changes in mean offshore wave conditions
Sustained changes in cross-section/ stability of mainland inlets	Medium/Long-term	Sea level rise, changes in mean offshore wave conditions, changes in annual riverflow
Chronic coastline recession (uninterrupted coasts)	Long-term	Sea level rise, changes in storm surge, changes in storm wave characteristics
Chronic coastline recession (inlet interrupted coasts)	Long-term	Sea level rise, changes in riverflow, changes in fluvial sand supply, changes in storm surge, changes in storm wave characteristics
Barrier Island thinning	Long-term	Sea level rise
Barrier rollover	Long-term	Sea level rise, changes in storm surge, changes in storm wave characteristics

*Time scale definitions: Episodic ~ hours-days, medium-term ~ year - decade, and long-term (~ decades - century).

Table 1 illustrates that climate change impacts on sandy coasts will be governed not only by SLR, but also by variations in wave conditions (mean and extreme), storm surges and riverflows, and combinations thereof. The fundamental question that needs to be answered then is *“how will the world’s coastlines respond to the integrated effect of climate change driven variations in mean sea level, storm surge, waves, and riverflows?”*. As is the case with any attempt to assess hazards that may occur in the future, numerical modelling provides the only avenue towards answering this question; but do we have the right models to do the job?

## Numerical Modelling of Climate Change Driven Coastal Change

At present, climate change impacts on coastal change are commonly estimated via: (a) one-dimensional, physically based, but simple models (e.g. Bruun Rule^26^); (b) highly scale-aggregated models with limited process descriptions (e.g. ASMITA^27^, CASCADE^28^); and (c) extensive time integration of micro-scale processes using process-based morphodynamic models^29–31^. For the convenience of the reader, all coastal change models mentioned above and below are listed by model category in Table 2.

**Table 2:** Coastal change models mentioned in this article (by model category).

Reference	Model name (where applicable)
***Simple physics based models***	
Bruun, P. Sea-Level Rise as a Cause of Shore Erosion. *J. Waterw. Harb. Div*. **88**, 117–132 (1962)	*Bruun Rule*
***Scale aggregated semi-empirical models***	
Stive, Marcel J. F., Wang, Z. Morphodynamic modelling of tidal basins and coastal inlets. in *Advances in Coastal Modelling* (ed. Lakhan, C.) 367–392 (Elsevier Science Publishers B.V, 2003)	*ASMITA*
Larson, M., Kraus, N. & Hanson, H. Simulation of Regional Longshore Sediment Transport and Coastal Evolution - The ‘Cascade’ Model. in *Proc 28th Int Coastal Eng Conf. American Society of Civil Engineers (ASCE)* 2612–2624 (ASCE, 2002)	*CASCADE*
***Behaviour oriented models***	
Dabees, M. & Kamphuis, J. ONELINE: Efficient Modeling of 3-D Beach Change. in *Proceedings of the 27th International Conference on Coastal Engineering* 2700–2713 (ASCE, 2000)	*ONELINE*
Hanson, H. *et al*. Modelling of Coastal Evolution on Yearly to Decadal Time Scales. *J. Coast. Res*. **19**, 790–811 (2003)	*GENESIS*
***Process based models***	
Ranasinghe, R. Assessing climate change impacts on open sandy coasts: A review. *Earth-Science Rev*. **160**, 320–332 (2016)	*Delft3D*
Roelvink, J. A. Coastal morphodynamic evolution techniques. *Coast. Eng*. **53**, 277–287 (2006)	
Dissanayake, D. M. P. K., Ranasinghe, R. & Roelvink, J. A. The morphological response of large tidal inlet/basin systems to relative sea level rise. *Clim. Change* **113**, 253–276 (2012)	
van der Wegen, M. Numerical modeling of the impact of sea level rise on tidal basin morphodynamics. *J. Geophys. Res. Earth Surf*. **118**, 447–460 (2013)	
Lesser, G. An approach to medium-term coastal morphological modeling. (UNESCO-IHE/Delft University of Technology, Netherlands, 2009)	
Duong, T. M., Ranasinghe, R., Luijendijk, A., Walstra, D. & Roelvink, D. Assessing climate change impacts on the stability of small tidal inlets: Part 1 - Data poor environments. *Mar. Geol*. **390**, 331–346 (2017)	
Duong, T. M. *et al*. Assessing climate change impacts on the stability of small tidal inlets: Part 2 - Data rich environments. *Mar. Geol*. **395**, 65–81 (2018)	
Luijendijk, A., Schipper, M. & Ranasinghe, R. Morphodynamic Acceleration Techniques for Multi-Timescale Predictions of Complex Sandy Interventions. *J. Mar. Sci. Eng*. **7**, 78 (2019)	
Ranasinghe, R. Assessing climate change impacts on open sandy coasts: A review. *Earth-Science Rev*. **160**, 320–332 (2016)	*Mike21*
Ranasinghe, R. Assessing climate change impacts on open sandy coasts: A review. *Earth-Science Rev*. **160**, 320–332 (2016)	*CMS*
Reniers, A. J. H. M., Thornton, E. B., Stanton, T. P. & Roelvink, J. A. Vertical flow structure during Sandy Duck: observations and modeling. *Coast. Eng*. **51**, 237–260 (2004)	*Quasi 3D –Delft3D*
***Reduced complexity models***	
Roscoe, K. L. & Diermanse, F. Effect of surge uncertainty on probabilistically computed dune erosion. *Coast. Eng*. **58**, 1023–1033 (2011)	N/A
Ranasinghe, R., Callaghan, D. & Stive, M. J. F. Estimating coastal recession due to sea level rise: beyond the Bruun rule. *Clim. Change* **110**, 561–574 (2012)	*PCR*
Ranasinghe, R., Duong, T. M., Uhlenbrook, S., Roelvink, D. & Stive, M. Climate-change impact assessment for inlet-interrupted coastlines. *Nat. Clim. Chang*. **3**, 83–87 (2013).	*SMIC*
Toimil, A., Losada, I. J., Camus, P. & Díaz-Simal, P. Managing coastal erosion under climate change at the regional scale. *Coast. Eng*. **128**, 106–122 (2017)	N/A
Ashton, A., Murray, A. B. & Arnoult, O. Formation of coastline features by large-scale instabilities induced by high-angle waves. *Nature* **414**, 296–300 (2001)	*CEM*
Ashton, A. D. & Murray, A. B. High-angle wave instability and emergent shoreline shapes: 1. Modeling of sand waves, flying spits, and capes. *J. Geophys. Res*. **111**, F04011 (2006)	
Ratliff, K. M. & Murray, A. B. Modes and emergent time scales of embayed beach dynamics. *Geophys. Res. Lett*. **41**, 7270–7275 (2014)	
Wolinsky, M. A. A unifying framework for shoreline migration: 1. Multiscale shoreline evolution on sedimentary coasts. *J. Geophys. Res*. **114**, F01008 (2009)	N/A
Rosati, J. D., Dean, R. G. & Walton, T. L. The modified Bruun Rule extended for landward transport. *Mar. Geol*. **340**, 71–81 (2013)	*Modified Bruun rule*
Durán Vinent, O. & Moore, L. J. Barrier island bistability induced by biophysical interactions. *Nat. Clim. Chang*. **5**, 158–162 (2015)	N/A
Vitousek, S., Barnard, P. L., Limber, P., Erikson, L. & Cole, B. A model integrating longshore and cross-shore processes for predicting long-term shoreline response to climate change. *J. Geophys. Res. Earth Surf*. **122**, 782–806 (2017)	*COSMOS*
Robinet, A., Idier, D., Castelle, B. & Marieu, V. A reduced-complexity shoreline change model combining longshore and cross-shore processes: The LX-Shore model. *Environ. Model. Softw*. **109**, 1–16 (2018)	*LX-SHORE*
Mendoza, E. & Jiminez, J. Storm-Induced Beach Erosion Potential on the Catalonian Coast. *J. Coast. Res*. 81–88 (2006)	N/A
Splinter, K. D. *et al*. A generalized equilibrium model for predicting daily to interannual shoreline response. *J. Geophys. Res. Earth Surf*. **119**, 1936–1958 (2014)	N/A
Larson, M., Palalane, J., Fredriksson, C. & Hanson, H. Simulating cross-shore material exchange at decadal scale. Theory and model component validation. *Coast. Eng*. **116**, 57–66 (2016)	*CSM*
Palalane, J. *et al*. Simulating cross-shore material exchange at decadal scale. Model application. *Coast. Eng*. **116**, 26–41 (2016)	

Straightforward applications of simple techniques such as the Bruun rule, while potentially being adequate for first-pass assessments at regional to global scale, are unlikely to produce results that are sufficiently reliable to support local scale coastal management/adaptation decisions with US $ billions at stake^32,33^. Highly aggregated models such as ASMITA and CASCADE essentially drive the models toward a prescribed end-state (i.e. equilibrium condition). Due to the empirically based (usually with data from one or two data rich locations) severe aggregation inherent in these models, they do not provide much insight on processes governing morphological evolution, and their general applicability is also somewhat tenuous. Attempts to date with fully process-based models (e.g. *Delft3D*, *Mike21*, *CMS*) forced with concurrent water level, wave, and riverflow forcing have only been able to produce accurate results for simulation lengths less than about 5 years^5,34–36^. Therefore, it appears that currently available modelling approaches are unable to provide sufficiently reliable predictions of integrated climate change impact on coastal change, and that new models underpinned by ‘out-of-the-box’ thinking are urgently needed.

As climate change impacts on sandy coasts will manifest themselves at various different spatio-temporal scales (~10 m to ~100 km and days to centuries; see Table 1), ideally what is required for climate change impact assessments is a multi-scale coastal change model that concurrently simulates the various physical processes occurring at different spatio-temporal scales, while also accounting for inter-scale morphodynamics.

### Process-based modelling

To simulate coastal hydrodynamics relevant for episodic (ST), medium-term, and long-term (LT) morphodynamics, a process-based multi-scale model needs to incorporate both cross-shore (vertically non-uniform) and longshore (mostly vertically uniform) hydrodynamics. Ideally, therefore, the model would need to be a process based model capable of simulating nearshore hydrodynamics in at least a quasi-3D fashion. Previous attempts at quasi-3D representation of nearshore coastal hydrodynamics have been successful (e.g^37^.), and therefore, this is not a major challenge. However, modelling morphological change that may occur under the combined forcing of waves and currents (including riverflow effects at coastal inlets), especially at time scales of more than a few years, still remains a significant challenge^34,38^. Although, there have been numerous attempts since the early 1990s to overcome this challenge, these have met with only partial success, at best^29,38–40^. The most recent attempt, which used a combination of parallel computing and wave input reduction techniques, achieved a 30-year morphodynamic simulation with combined wave-tide forcing^41^, with a computing time of 5–19 days (depending on the parallel computing/input reduction combination used). While this is a huge improvement from what was possible 10 years ago, achieving a 100 year wave-tide-riverflow forced simulation within a few minutes (or hours) using traditional process-based models still seems very far away.

Perhaps a completely different approach is required to solve this problem. For example, the solution could lie in a novel concept in which morphological change is simulated using a non-gridded technique. In such a model, for instance, a traditional computational grid may still be used to compute time varying quasi-3D nearshore water level, velocity and transport fields, but these quantities would then be spatially and temporally aggregated in areas of interest where potentially mobile morphological features exist (e.g. sand bars, channels, mounds, ebb/flood deltas etc.). These aggregated hydrodynamic forcing fields may subsequently be used, in combination with an appropriate morphodynamic acceleration factor, to rapidly simulate the spatio-temporal evolution of only the morphological features of interest over a few tidal cycles. If successful, such an approach would enable morphodynamic simulations that are much faster, and consequently much longer, than what the present state-of-the-art would allow.

### Reduced complexity modelling

While new ideas and increasing computational power might enable ~100-year long process-based model simulations in the future, still, their inherent slowness, will probably render this type of models unwieldy for the multiple simulations (~1000) required to derive probabilistic estimates of coastal change; a mandatory requirement of emerging risk informed coastal zone management/planning frameworks^42,43^. An effective approach to circumnavigate this problem is to develop physics based, yet simple and fast numerical models known as reduced complexity models. This approach adopts simplified descriptions of fundamental system physics and delivers estimates of system response to forcing. It is a well-grounded and fast approach that lends itself to multiple simulations (thousands of simulations in minutes), enabling probabilistic estimates of system response.

While a few such reduced complexity numerical models have been developed since the turn of the century^44–47^, no concerted efforts have yet been made to develop a reduced complexity model that is capable of providing rapid, probabilistic estimates of coastal change resulting from the integrated effect of climate change driven variations in coastal forcing. Such an attempt, which would undoubtedly be a challenging undertaking, could for example follow the basic approach outlined below.

Relevant concepts adopted in existing non-process based LT coastal evolution models (e.g^48–55^.) could be used to develop a reduced complexity model to obtain rapid, probabilistic estimates of future LT nearshore morphological change. This model can then potentially be combined with an existing ST reduced complexity models, such as the Probabilistic Coastal Recession (PCR) model^45^, which provides probabilistic estimates of contemporary and/or future ST storm erosion volumes. Concepts adopted in existing non-process based models of ST coastal change (e.g^56–59^.) may also be strategically used in the model development depending on local geomorphic conditions and/or the target coastline indicator (e.g. MSL contour, toe/top of dune, vegetation line).

The main result that can be expected from the application of such an LT/ST integrated, 2D reduced complexity coastal change model is a series of coastline positions (alongshore) with a range of exceedance probabilities (e.g. 0.9, 0.5, 0.1, 0.01) for every year of the simulation. These probabilistic results could then be combined with, for e.g. spatial maps of property value to derive economic risk maps.

It is important to note that the confidence with which any of the above discussed novel modelling approaches can be applied to address real-world situations depends very much on rigorous model validation against field measurements. The non-availability of (or lack of free access to) long term morphological and hydrodynamic data has been for decades a frustrating bottleneck in terms of achieving robust validation of, especially, longer term coastal change models. However, the recent emergence of open source satellite image based global data sets of coastal morphology and topography^24,60–64^ and the general worldwide trend towards open source *in-situ* data (e,g. EMODNET, CEFASWavenet, SISMER, SHOM, Open Earth, DUCK FRF, Narrabeen-Collaroy) represents a step-change in the availability of/access to long term data, greatly improving opportunities for the validation of long term coastal change models.

While the economic damage (consequence) that can be caused by climate change driven coastal change (hazard) can be very high, foregoing land-use opportunities in coastal regions is also costly (opportunity cost), with both sides of the equation depending not only on climate change impacts but also on economic considerations such as future changes in coastal property values, and return on investments in the coastal zone etc^65^. Developing effective policies and strategies for future coastal land-use planning purposes is therefore a delicate balancing act. Quantitative coastal risk assessments are also invaluable to the insurance and re-insurance industries for determining optimal insurance premia, with follow-on effect on coastal property values, and subsequently on the value-at-risk^65–70^. Projections of future coastal change provided by a new generation of multi-scale, probabilistic coastal change models such as those discussed above will readily support comprehensive coastal risk assessment and optimisation, enabling risk informed, climate proof adaptation measures that optimises the balance between risk and reward.

## References

[1] McGranahan G, Balk D, Anderson B (2007). The rising tide: assessing the risks of climate change and human settlements in low elevation coastal zones. Environ. Urban..

[2] Hinkel J (2013). A global analysis of erosion of sandy beaches and sea-level rise: An application of DIVA. Glob. Planet. Change.

[3] Wong, P. *et al*. Coastal Systems and Low-Lying Areas Coordinating Lead Authors: Lead Authors: Review Editors. in *Climate Change 2014: Impacts,Adaptation, and Vulnerability. Part A: Global and Sectoral Aspects. Contribution of Working Group II to the Fifth Assessment Report of the Intergovernmental Panel on Climate Change* (eds. Field, C. B. *et al*.) 361–409 (Cambridge University Press, 2014). 10.1017/CBO9781107415379.010

[4] Brown S (2014). Shifting perspectives on coastal impacts and adaptation. Nat. Clim. Chang..

[5] Ranasinghe R (2016). Assessing climate change impacts on open sandy coasts: A review. Earth-Science Rev..

[6] Vousdoukas MI (2018). Global probabilistic projections of extreme sea levels show intensification of coastal flood hazard. Nat. Commun..

[7] The World Bank. *World Development Report 2010: Development and Climate Change*. (2010).

[8] Besset, M., Anthony, E. J. & Bouchette, F. Multi-decadal variations in delta shorelines and their relationship to river sediment supply: An assessment and review. *Earth-Science Rev*. in press (2019).

[9] Ranasinghe R, Wu CS, Conallin J, Duong TM, Anthony EJ (2019). Disentangling the relative impacts of climate change and human activities on fluvial sediment supply to the coast by the world’s large rivers: Pearl River Basin, China. Sci. Rep..

[10] Stern, N. *The Economics of Climate Change*. 10.1017/CBO9780511817434 (Cambridge University Press, 2007).

[11] Arkema KK (2013). Coastal habitats shield people and property from sea-level rise and storms. Nat. Clim. Chang..

[12] Kron W (2013). Coasts: the high-risk areas of the world. Nat. Hazards.

[13] Brown S, Nicholls RJ, Lowe JA, Hinkel J (2016). Spatial variations of sea-level rise and impacts: An application of DIVA. Clim. Change.

[14] McNamara DE, Keeler A (2013). A coupled physical and economic model of the response of coastal real estate to climate risk. Nat. Clim. Chang..

[15] Johnson JM (2015). Recent shifts in coastline change and shoreline stabilization linked to storm climate change. Earth Surf. Process. Landforms.

[16] Hallegatte S, Green C, Nicholls RJ, Corfee-Morlot J (2013). Future flood losses in major coastal cities. Nat. Clim. Chang..

[17] Church, J. A. *et al*. *Sea level change*. *Climate Change 2013: The Physical Science Basis. Contribution of Working Group I to the Fifth Assessment Report of the Intergovernmental Panel on Climate Change*. 10.1017/CB09781107415315.026 (2013).

[18] Hemer MA, Fan Y, Mori N, Semedo A, Wang XL (2013). Projected changes in wave climate from a multi-model ensemble. Nat. Clim. Chang..

[19] Mentaschi L, Vousdoukas MI, Voukouvalas E, Dosio A, Feyen L (2017). Global changes of extreme coastal wave energy fluxes triggered by intensified teleconnection patterns. Geophys. Res. Lett..

[20] Mori N (2019). Future changes in extreme storm surges based on mega-ensemble projection using 60-km resolution atmospheric global circulation model. Coast. Eng. J..

[21] Collins, M. *et al*. Long Term Climate Change: Projections, Commitments and Irreversibility. in *Climate Change 2013: The Physical Science Basis. Contribution of Working Group I to the Fifth Assessment Report of the Intergovernmental Panel on Climate Change* (eds. Stocker, T. F. *et al*.) 1029–1136 (Cambridge University Press, 2013).

[22] Nicholls RJ, Cazenave A (2010). Sea-level rise and its impact on coastal zones. Science.

[23] Cazenave A, Cozannet G (2014). Le. Sea level rise and its coastal impacts. Earth’s Futur..

[24] Luijendijk A (2018). The State of the World’s Beaches. Sci. Rep..

[25] Small C, Nicholls RJ (2003). A Global Analysis of Human Settlement in Coastal Zones. J. Coast. Res..

[26] Bruun P (1962). Sea-Level Rise as a Cause of Shore Erosion. J. Waterw. Harb. Div..

[27] Stive, M. J. F. & Wang, Z. Morphodynamic modelling of tidal basins and coastal inlets. in *Advances in* Coastal Modelling (ed. Lakhan, C.) 367–392 (Elsevier Science Publishers B.V, 2003).

[28] Larson, M., Kraus, N. & Hanson, H. Simulation of Regional Longshore Sediment Transport and Coastal Evolution - The ‘Cascade’ Model. in *Proc 28th Int Coastal Eng Conf. American Society of Civil Engineers (ASCE)* 2612–2624 (ASCE, 2002).

[29] Roelvink JA (2006). Coastal morphodynamic evolution techniques. Coast. Eng..

[30] Dissanayake DMPK, Ranasinghe R, Roelvink JA (2012). The morphological response of large tidal inlet/basin systems to relative sea level rise. Clim. Change.

[31] van der Wegen M (2013). Numerical modeling of the impact of sea level rise on tidal basin morphodynamics. J. Geophys. Res. Earth Surf..

[32] Cooper JAG, Pilkey OH (2004). Sea-level rise and shoreline retreat: time to abandon the Bruun Rule. Glob. Planet. Change.

[33] Ranasinghe R, Stive MJF (2009). Rising seas and retreating coastlines. Clim. Change.

[34] Lesser, G. An approach to medium-term coastal morphological modeling. (UNESCO-IHE/Delft University of Technology, Netherlands, 2009).

[35] Duong TM, Ranasinghe R, Luijendijk A, Walstra D, Roelvink D (2017). Assessing climate change impacts on the stability of small tidal inlets: Part 1 - Data poor environments. Mar. Geol..

[36] Duong TM (2018). Assessing climate change impacts on the stability of small tidal inlets: Part 2 - Data rich environments. Mar. Geol..

[37] Reniers AJHM, Thornton EB, Stanton TP, Roelvink JA (2004). Vertical flow structure during Sandy Duck: observations and modeling. Coast. Eng..

[38] de Vriend HJ (1993). Medium-term 2DH coastal area modelling. Coast. Eng..

[39] Dabees, M. & Kamphuis, J. ONELINE: Efficient Modeling of 3-D Beach Change. in *Proceedings of the 27th International Conference on Coastal Engineering* 2700–2713 (ASCE, 2000).

[40] Hanson H (2003). Modelling of Coastal Evolution on Yearly to Decadal Time Scales. J. Coast. Res..

[41] Luijendijk A, Schipper M, Ranasinghe R (2019). Morphodynamic Acceleration Techniques for Multi-Timescale Predictions of Complex Sandy Interventions. J. Mar. Sci. Eng..

[42] Wainwright DJ (2015). Moving from deterministic towards probabilistic coastal hazard and risk assessment: Development of a modelling framework and application to Narrabeen Beach, New South Wales, Australia. Coast. Eng..

[43] Jongejan R, Ranasinghe R, Wainwright D, Callaghan DP, Reyns J (2016). Drawing the line on coastline recession risk. Ocean Coast. Manag..

[44] Roscoe KL, Diermanse F (2011). Effect of surge uncertainty on probabilistically computed dune erosion. Coast. Eng..

[45] Ranasinghe R, Callaghan D, Stive MJF (2012). Estimating coastal recession due to sea level rise: beyond the Bruun rule. Clim. Change.

[46] Ranasinghe R, Duong TM, Uhlenbrook S, Roelvink D, Stive M (2013). Climate-change impact assessment for inlet-interrupted coastlines. Nat. Clim. Chang..

[47] Toimil A, Losada IJ, Camus P, Díaz-Simal P (2017). Managing coastal erosion under climate change at the regional scale. Coast. Eng..

[48] Ashton A, Murray AB, Arnoult O (2001). Formation of coastline features by large-scale instabilities induced by high-angle waves. Nature.

[49] Ashton AD, Murray AB (2006). High-angle wave instability and emergent shoreline shapes: 1. Modeling of sand waves, flying spits, and capes. J. Geophys. Res..

[50] Wolinsky MA (2009). A unifying framework for shoreline migration: 1. Multiscale shoreline evolution on sedimentary coasts. J. Geophys. Res..

[51] Rosati JD, Dean RG, Walton TL (2013). The modified Bruun Rule extended for landward transport. Mar. Geol..

[52] Ratliff KM, Murray AB (2014). Modes and emergent time scales of embayed beach dynamics. Geophys. Res. Lett..

[53] Durán Vinent O, Moore LJ (2015). Barrier island bistability induced by biophysical interactions. Nat. Clim. Chang..

[54] Vitousek S, Barnard PL, Limber P, Erikson L, Cole B (2017). A model integrating longshore and cross-shore processes for predicting long-term shoreline response to climate change. J. Geophys. Res. Earth Surf..

[55] Robinet A, Idier D, Castelle B, Marieu V (2018). A reduced-complexity shoreline change model combining longshore and cross-shore processes: The LX-Shore model. Environ. Model. Softw..

[56] Mendoza, E. & Jiminez, J. Storm-Induced Beach Erosion Potential on the Catalonian Coast. *J. Coast. Res*. 81–88 (2006).

[57] Splinter KD (2014). A generalized equilibrium model for predicting daily to interannual shoreline response. J. Geophys. Res. Earth Surf..

[58] Larson M, Palalane J, Fredriksson C, Hanson H (2016). Simulating cross-shore material exchange at decadal scale. Theory and model component validation. Coast. Eng..

[59] Palalane J (2016). Simulating cross-shore material exchange at decadal scale. Model application. Coast. Eng..

[60] Mentaschi L, Vousdoukas MI, Pekel J-F, Voukouvalas E, Feyen L (2018). Global long-term observations of coastal erosion and accretion. Sci. Rep..

[61] Athanasiou P (2019). Global distribution of nearshore slopes with implications for coastal retreat. Earth Syst. Sci. Data.

[62] Yamazaki D (2017). A high-accuracy map of global terrain elevations. Geophys. Res. Lett..

[63] Farr TG (2007). The Shuttle Radar Topography Mission. Rev. Geophys..

[64] Kulp SA, Strauss BH (2019). New elevation data triple estimates of global vulnerability to sea-level rise and coastal flooding. Nat. Commun. 2019 101.

[65] Jongejan RB, Ranasinghe R, Vrijling JK, Callaghan D (2011). A risk-informed approach to coastal zone management. Aust. J. Civ. Eng..

[66] Ranasinghe, R. & Callaghan, D. P. Assessing storm erosion hazards. in *Coastal* Storms (eds. Ciavola, P. & Coco, G.) 241–256 (Springer, 2017).

[67] Rumson AG, Hallett SH (2019). Innovations in the use of data facilitating insurance as a resilience mechanism for coastal flood risk. Sci. Total Environ..

[68] Botzen WJW, Van Den Bergh JCJM (2008). Insurance Against Climate Change and Flooding in the Netherlands: Present, Future, and Comparison with Other Countries. Risk Anal..

[69] Filatova T, Mulder JPM, van der Veen A (2011). Coastal risk management: How to motivate individual economic decisions to lower flood risk?. Ocean Coast. Manag..

[70] Jongman B, Koks EE, Husby TG, Ward PJ (2014). Increasing flood exposure in the Netherlands: implications for risk financing. Nat. Hazards Earth Syst. Sci..

